# Validation of a German short version of the Attitudes towards Patient Safety Questionnaire (G-APSQshort) for the measurement of undergraduate medical students' attitudes to and needs for patient safety

**DOI:** 10.3205/zma001085

**Published:** 2017-02-15

**Authors:** Jan Kiesewetter, Moritz Kager, Martin R. Fischer, Isabel Kiesewetter

**Affiliations:** 1 Klinikum der LMU München, Institut für Didaktik und Ausbildungsforschung in der Medizin, München, Germany; 2 Klinikum der LMU München, Klinik für Anästhesiologie, München, Germany; 3 Klinikum der LMU München, Klinik für Palliativmedizin, München, Germany

## Abstract

**Introduction:** Topics of patient safety are being taught increasingly within medical eudcation. To date, however, there is no suitable means of measuring the status quo of medical students’ attitudes towards patient safety in German-speaking Europe. The German validation of a short version of the Attitude towards Patient Safety Questionnaire (G-APSQ*short*) is meant to fill this gap with the aid of two validation studies.

**Methods: **In Study 1, item and reliability analyses were used to examine internal consistency as well as factorial structure. In Study 2, the measurement sensitivity of the G-APSQ*short* in detecting changes in attitudes was assessed.

**Results: **Study 1 comprised N=83 participants (M=23.16 years; 21 female). Adequate internal consistency (Cronbach’s α=.722-.903) was reached in 6 of the seven subscales. The factor analysis showed that the six extracted factors matched the theoretically conceived subscales.

Study 2 comprised N=21 participants (M=26 years; 11 female). A multivariate analysis of variance showed that the differences before and after a short-term intervention were significant with medium effect size (F(1;16)=6.675; p<.05; η^2^=.29).

**Discussion: **In six subscales, the G-APSQ*short* can be considered valid in respect to measuring point and change. It is hoped that regular and concerted implementation of measuring instruments such as the G-APSQ*short* will help to develop a common ground for data comparison among many different German-speaking medical faculties.

## Zusammenfassung

**Hintergrund: **Zunehmend sollen Themen der Patientensicherheit auch im Rahmen des Medizinstudiums gelehrt werden. Für eine Bedarfsanalyse existiert im deutschsprachigen Raum jedoch bisher kein geeignetes Messinstrument zur Erhebung des Status Quo von Einstellungen Medizinstudierender zum Thema Patientensicherheit. Die deutsche Validierung einer Kurzversion des Attitudes towards Patient Safety Questionnaire (G-APSQ*short*), soll diese Lücke anhand von zwei Validierungsstudien schließen.

**Methode:** In Studie 1 wird mittels Item- und Reliabilitätsanalysen die interne Konsistenz, sowie die faktorielle Struktur überprüft. In Studie 2 wird die Messsensitivität des G-APSQ*short* zum Nachweis von Einstellungsveränderungen eingeschätzt.

**Ergebnisse: **An Studie 1 nahmen N=83 Teilnehmer (M=23,16 Jahre; 21 Weiblich) teil. Von den insgesamt sieben Subskalen konnte bei sechs Subskalen ausreichende interne Konsistenz (Cronbachs α=.722-.903) erreicht werden. Die Faktorenanalyse ergab, dass die sechs extrahierten Faktoren mit den theoretisch konzipierten Subskalen übereinstimmen.

An Studie 2 nahmen N=21 Teilnehmer (M=26 Jahre; 11 weiblich) teil. Eine Multivariate Varianzanalyse ergab, dass die Unterschiede vor- zu nach einer Kurzzeitintervention mit mittelgroßem Effekt signifikant geworden sind (F(1;16)=6,675; p<.05; η^2^=.29). Die Reliabilität der Differenzen der Messzeitpunkte war mit Cronbachs α=.615 ausreichend hoch.

**Diskussion:** Der G-APSQ*short* kann in sechs Subskalen als messzeitpunkt- und veränderungsvalide gelten. Es ist zu hoffen, dass durch den regelmäßigen und konzertierten Einsatz von Messinstrumenten wie dem G-APSQshort eine gemeinsame Datenbasis vieler verschiedener deutschsprachiger medizinischer Fakultäten entsteht.

## Introduction

In recent years, issues of medical errors and patient safety have increasingly gained in significance in the general public as well as in the fields of medicine and of healthcare policy making [1], [2], [3], [4]. The German Coalition for Patient Safety *(Aktionsbündnis Patientensicherheit e.V.)*, founded in 2005, the German Patient Rights Act from 2013, the increasing number of error-reporting systems established by medical professionals, as well as numerous reports in popular media all serve to underscore this development.

To date, little attention has been paid to training and preparing doctors in these regards, despite the fact that they, as prominent providers of healthcare, are not only significantly involved in the occurrence and prevention of medical errors but are also significantly affected by their consequences. Various international committees have demanded the early integration of corresponding medical-error and patient-safety educational structures in the training, continuing education and further education of medical professionals [5], [6], [7]. The guidelines presented by the WHO World Alliance for Patient Safety in 2008 for the development of corresponding error-management and patient-safety curricula were designed explicitly as a substantive basis for medical faculties [6]. The guidelines contain detailed, content-related information on all subject areas in the realm of patient safety as well as instruction for the successful implementation of new curricula. Thus far, very few of the implemented educational formats addressing errors in medicine and patient safety have been described in international publications. Only a small number of educational formats incorporate the WHO guidelines in their application, and their structure and content display an expansive range in their implementation [8]. 

In Germany, first steps in the development of corresponding curricular structures are being taken, and the German National Learning Objectives Catalogue [NKLM] has also included the subject of patient safety in its material [9], [10]. In 2015, the German Medical Education Association’s Committee for Patient Safety and Error Management published. The Learning Objective Catalogue for Patient Safety in Undergraduate Medical Education, representing a consensus on the most important learning objectives for patient safety [11]. The Attitudes towards Patient Safety Questionnaire (APSQ) was developed and validated for English-speaking regions in order to measure the attitudes of medical students [12]. The original version identified nine subscales:

- Patient safety training received
- Error reporting confidence
- Working hours as error cause
- Error inevitability
- Professional incompetence as error cause
- Disclosure responsibility
- Team functioning
- Patient’s role in error
- Importance of patient safety in the curriculum

In the original scale’s criterion validation, seven subscales could differentiate between novice students and experienced tutors. The scales are reliable, with a Cronbach’s alpha of .63 to .82. The 26 items are distributed among 9 subscales with 2 to 4 items each. They comprise questions requiring responses from “strongly agree” to “strongly disagree”. The following is an example from the subscale Error Reporting Confidence: “I would feel comfortable reporting any errors I had made, no matter how serious the outcome had been for the patient.” [12].

The authors recommend further validation studies of retest reliability and predictive validity. The reliability of change of the APSQ has yet to be verified. 

Such a measure can be used to track individual changes throughout the course of studies, allowing for targeted training interventions and evaluation of their success. Furthermore, comparative measures of different training or curricular modules or even entire curricula on the subject of patient safety [13] are possible. Fields of application for the questionnaire in interprofessional and post-graduate education and training are also being discussed [14]. So far, results in the United States offer a mixed picture of medical students’ attitudes at the beginning of their studies: On the one hand, medical students demonstrate a positive attitude, conducive to patient safety. On the other hand, confusion and uncertainty prevail concerning error-reporting procedures, error communication and the consequences of errors for the individual [8]. To date, little is known regarding attitudes towards patient safety during the course of curricula and at the end of studies. 

At the moment, there is no instrument in German-speaking countries for a systematic gathering of data from the students’ perspective on their needs or on their attitude toward and handling of errors in treatment. The present study should serve to close this gap with the German-language validation of a short version of the Attitudes towards Patient Safety Questionnaire (G-APSQ*short*). This instrument was selected as the most widely validated measure of its kind. The validation sought here is one of construct validity, meaning that the instrument is being examined for its ability to measure the targeted feature of attitudes towards patient safety (=construct). 

## Aims

The present article examines two aspects of the APSQ:

1. Development and test-theoretical examination of a German-language short version of the APSQ (G-APSQ*short*) for measuring attitudes towards patient safety using item and reliability analyses for internal consistency, as well as the examination of factorial structure, that is, the examination of which subscales are also interpretable in German-speaking regions.
2. Examination of the reliability of change of the G-APSQ*short* in detecting changes in attitudes towards patient safety through short-term intervention.

## Methods

Two studies were designed to validate the questionnaire. In Study 1, the internal consistency and factorial structure of the questionnaire were examined using a sample of medical students (N=83). In Study 2, a smaller sample (N=21) of medical students was used to test the reliability of change of the scales that proved valid in Study 1. Consent was granted by the responsible medical faculty ethics commission for both studies. All data was gathered anonymously. 

### Translation

The translation of the questionnaire into German according to international standards preceded both studies [15]. Initially, two colleagues translated all of the items from English into German independently from each other; variances between the two translations were then discussed, and an agreement was reached on a mutual version. This first German version was then translated back into English by a third colleague whose native language is English. The resulting back-translation version was then compared to the English-language original version, showing minimal deviations which were resolved in communicative validation between the three colleagues and then incorporated in the final validation version of the questionnaire.

#### Pilot test

The pilot test of the questionnaire took place in the span of one week with n=22 medical students. The students gave feedback on the functionality and comprehensibility of the questions and were not permitted to take part in the ensuing study sample. 

#### Form

Analogous to the original version, the individual questions were scored on a Likert scale of 1 (strongly disagree) to 7 (strongly agree). These measures assured the best comprehensibility and the greatest conformity with the original questionnaire. 

#### Implications from the pilot test

**Figure 1:**
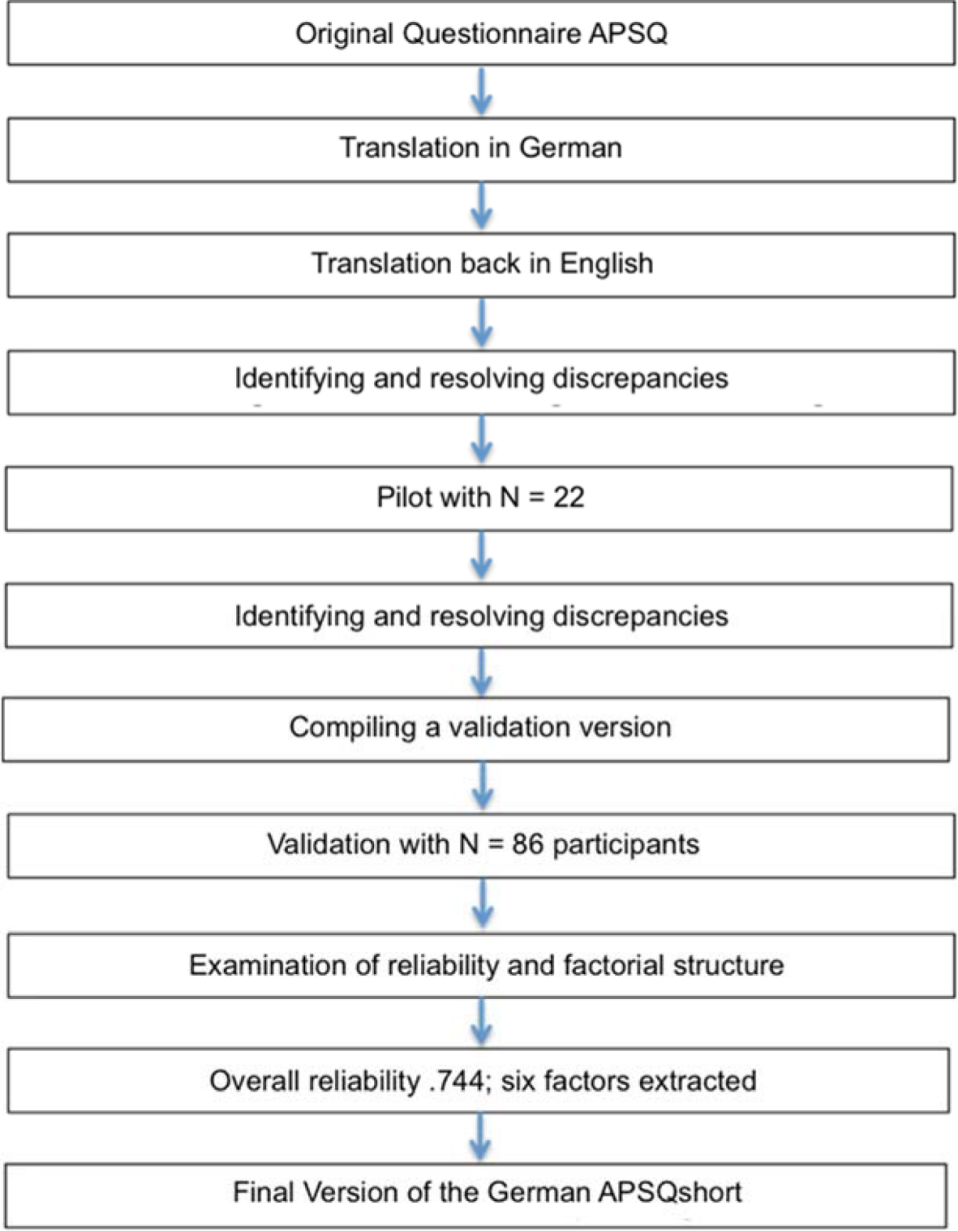
schematic overview of the creation of the final version of the G-APSQ*short*

During both the translation and the pilot test, the formulation of the items in accordance with the original wording in the subscales “disclosure responsibility” and “team functioning” proved to be very challenging. The translations were consistent with the original scales in the translation/back-translation step, but this resulted in a loss of the possibility for sensible differentiation in answerability. Ceiling effects arose from these items in the pilot test – all of the students assessed the items with full points. Because these were exactly the subscales that did not differentiate between the students and tutors in the original validation study, the authors chose to further validate a German short version of the APSQ (German APSQ short; G-APSQ*short*) (see Figure 1 (Fig. 1)).

#### Data collection

##### Study 1

The first phase of the questionnaire validation was performed with a sample drawn from among all medical students participating in the national conference of the German Medical Students‘ Association (*bvmd*) from November 30 to December 2, 2012. In total, approximately 200 students from all German medical faculties took part in the conference. The questionnaire was disseminated among the students during registration. Completed questionnaires could be returned to a centrally located box over the course of the weekend. As an incentive, twenty book vouchers with a value of ten euro each were raffled. 

##### Study 2

In curriculum development and evaluation, not only cross-sectional data concerning medical students’ attitudes towards error and patient safety are of interest, but also changes in these attitudes through relevant interventions for the purpose of education in patient safety [14]. For this reason, Study 2 was conducted in order to evaluate the validity of change of the G-APSQ*short*. To this end, data was gathered from a total of 21 students at the beginning and at the end of the one-week summer school “Errors in Medicine” in August 2013. During this week, the participants received intensive instruction and ensuing discussion opportunity in the principles of error theory, in legal, ethical and philosophical aspects of errors in medicine as well as in practical handling of diagnostic and treatment errors. 

#### Data analysis

The data for both studies was analyzed using SPSS Version 20.0. All reported significant data relate to an alpha error level of p=.05. All of the items were coded according to the original; higher values signify an attitude that would be beneficial to patient safety.

In Study 1, the reliability was first checked using Cronbach’s alpha. Subsequently, testing of the preconditions for a factor analysis was conducted using two statistical procedures for estimating the correlation of the items to each other. More precisely, the adequacy of the sample was assessed by means of the Kaiser-Meyer-Olkin (KMO) test, and the sphericity was examined using Bartlett’s test. The latter tests the data to determine whether they come from one population or are all uncorrelated. The KMO test indicates the proportion of variance among individual variables that is not explicable by other variables [16]. The factorial structure was examined by means of explorative factor analysis (maximum likelihood, with varimax rotation and Kaiser normalization).

In Study 2, a multivariate analysis of variance (MANOVA) was conducted for the entire scale. Within the MANOVA, the Pillai’s Trace Test was interpreted. LSD (Least Significant Difference) tests were used to clarify post-hoc interactive effects. For Study 2, the reliability of change (measuring point 2 – measuring point 1) was assessed using Cronbach’s α. 

## Results

### Study 1 

The sample included in Study 1 comprised N=83 data sets (M=23.16 years; SD=2.72; 46 female). The participants represented all of the three stages of medical study in Germany (pre-clinical N=17; clinical N=61; practical year N=5). Of the total of seven subscales, six subscales reached adequate reliability (Cronbach’s α=.722 - .903). Four items from the subscale “professional incompetence as error cause” had to be removed from the final questionnaire due to insufficient conformity to all of the other subscales. Table 1 (Tab. 1) gives an overview of the reliabilities. The entire scale showed a reliability of Cronbach’s α =.744. 

The Kaiser-Meyer-Olkin criterion was at 0.618, thus above the limit of 0.6 [17], and Bartlett’s sphericity test also proved significant (Chi^2^_(df=210)_=.680-.566;p<.001). The factorial structure resulted in six extracted factors (factor loading > .40) which agreed with the theoretically conceived subscales (“patient safety training received”, “error reporting confidence”, “working hours as error cause”, “error inevitability”, “patient’s role in error”, “importance of patient safety in the curriculum”). Altogether, the subscales explain 52.91% of the total variance. The extracted factors as well as the explained variance and descriptive statistics can be found in Table 1 (Tab. 1). The final G-APSQ*short* questionnaire comprises 14 items for six subscales and can be found in Attachment 1 of this article. For those interested, we have illustrated the initial eigenvalues of the items and the rotated loading matrix in Attachments 2 and Attachment 3 of this article. 

#### Study 2

The N=21 participants (11 female) came from ten different German medical faculties. The average age was 26 years. 

**Figure 2:**
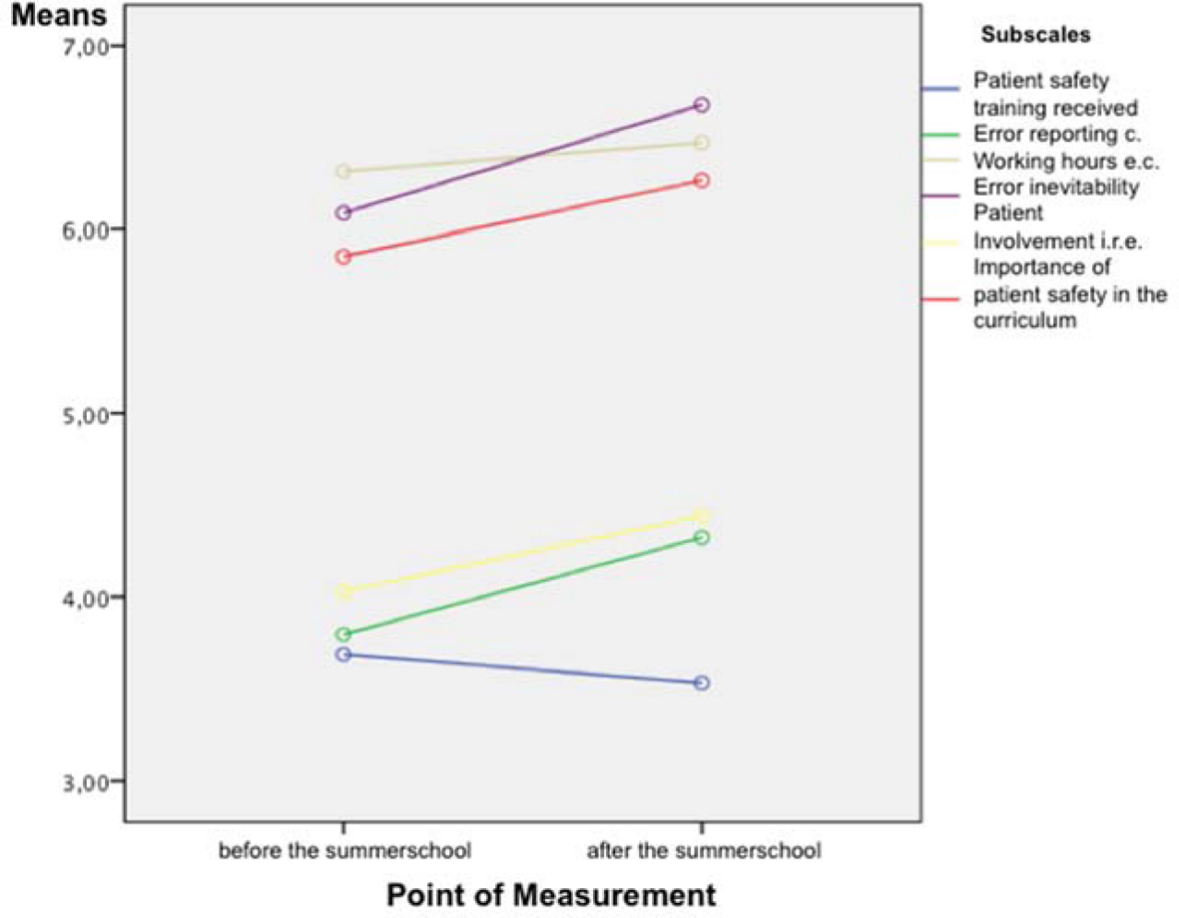
Changes in individual scales of the G-APSQ*short* during the summer school “Errors in Medicine”

The G-APSQ*short* yields a highly differentiated result on the change in attitude over the week concerning the subject. For the reliability of change Cronbach’s α=.615 was sufficiently sensitive for variance. The multivariate analysis of variance demonstrated that the differences between before and after the summer school had become significant with medium size effect (F(1;16)=6,675; p<.05; η^2^=.29). As can be seen in Figure 2 (Fig. 2), all of the scales contributed to this effect, with the exception of the scale “patient safety training received”. The LSD post hoc on interactive effects of measuring point and subscale is significant for six subscales (see Table 2 (Tab. 2)). The descriptive statistics for the subscales can be found in Table 2 (Tab. 2).

## Discussion

The German short version of the Attitudes towards Patient Safety Questionnaire can be deemed valid in six subscales. Despite a low number of overall items, six of the subscales already validated in the original version were successfully validated. The subscale “professional incompetence as error cause” could not be validated. Further assessment of the formulation may be necessary here. The reliability of the individual subscales can be qualified as exceptionally good. Favorably, despite a high reliability for one point of measurement, the reliability of change can also be considered as very good [16]. It is not surprising that the scale “patient safety training received” did not differ in comparison before and after the summer school, since it simply queried instruction during the course of undergraduate medical eduction (which the summer school was not a part of). The results of our study show great similarity with those of other international measurements regarding patient safety [8], [12]. Medical students‘ attitudes towards patient safety are principally positive (students recognize working hours as a source of errors, are conscious of the inevitability of errors, desire more instruction in patient safety), their confidence in reporting errors is, however, comparatively low, and the role of the patient in clarifying error is more or less unclear to them. 

Because of their use in the evaluation of interventions for the improvement of patient safety, the verified reliability of change is particularly important for measurement instruments regarding attitudes. These interventions could be applied both in smaller interventions, such as courses or workshops (as implemented by Jha [14], for example), and in entire curricula (as urged by Armitage et al. [13]). It should be noted that, for now, the subscales apply exclusively to medical students. Applications outside of this sample (e.g., residents, nurses) would appear advantageous. The G-APSQshort would, however, need to be revalidated beforehand.

### Limitations

The G-APSQ*short* is a short, written measurement instrument regarding medical students’ attitudes towards patient safety and treatment errors. The sample of N=83 simply represents a cross section of different medical faculties. Although this ensures independence from individual faculties (i.e., of which some of them might have integrated the subject of patient safety particularly well or particularly poorly in their curriculum), an effective representation of all medical students in Germany is not provided. Furthermore, it can be presumed that exceptionally dedicated students attended the bvmd conference. This limitation parallels that of the original study. 

The measurement of change presented here was taken on a small sample with very condensed instruction in a short period of time. Whether or not this instrument is valid for implementation in measurement of change over longer periods of time is ultimately not evidenced. Nonetheless, the medium effect size of the multivariate analysis of variance, as well as the sufficiently high reliability of change indicate that changes can be adequately depicted. A measurement of change over a longer period to strengthen the validation of the instrument would be advisable.

In the German short version, the measurement instrument only has two to three items per subscale, a range deemed insufficient by the authors of the original instrument. Nevertheless, the internal consistency of both the original instrument and the German short version rated as satisfactory. Only further research can indicate to what extent differentiated assertions can be made at the subscale level with the G-APSQ*short* or if extension of the individual subscales is necessary.

## Conclusions

The present measurement instrument can be regarded as validated for comparison of different faculties and for the evaluation of short-term intervention methods. Consisting of 14 items, it is relatively brief and clearly formulated. In light of advancing curriculum development, the authors hope that the G-APSQ*short* will be used as a measurement instrument for national and international comperability. In German-speaking Europe, the systematic development and collection of reliable data on patient safety is in its incipient stages. It is hoped that regular and concerted implementation of measurement instruments like the G-APSQ*short* will facilitate the emergence of a common ground for data comparison for many different medical faculties. 

## Acknowledgements

The authors wish to thank the Volkswagen Foundation for their financial support of the summer school. 

Additional thanks go to the German Medical Students’ Association (*bvmd*) for their support in data collection. 

## Competing interests

The authors declare that they have no competing interests.

## Supplementary Material

German Short Version of the Attitudes to Patient Safety Questionnaire G-APSQshort

Factor loadings of items and their distribution on the 6 factors

Eigenvalues of the items before rotation

## Figures and Tables

**Table 1:**
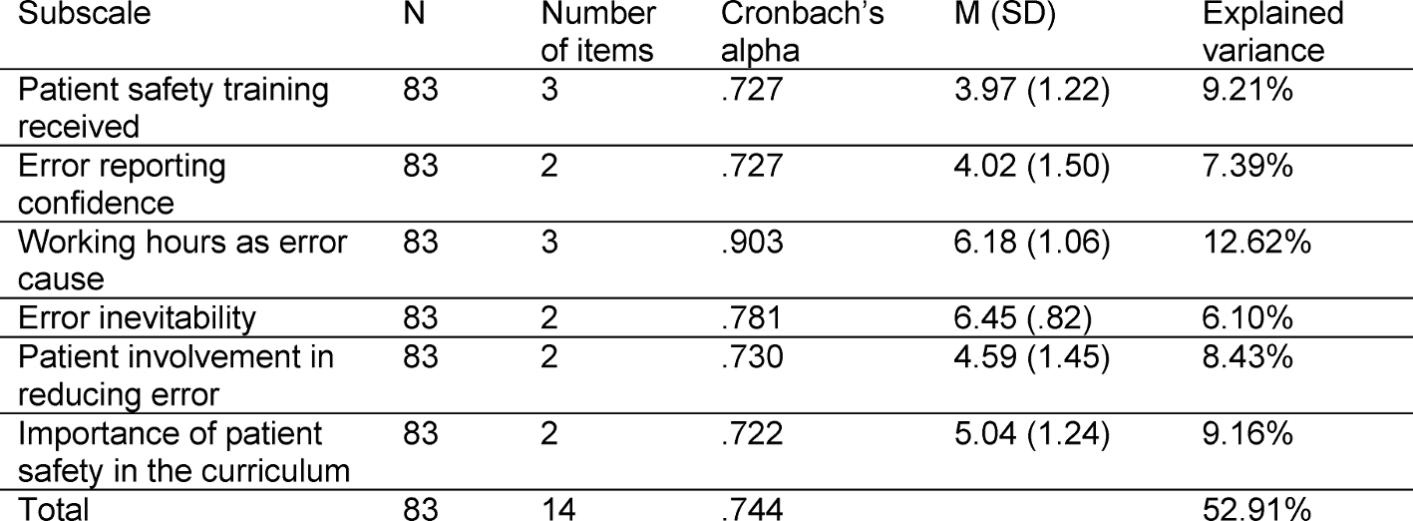
Reliability, descriptive statistics and variance clarification of the scales measured as reliable.

**Table 2:**
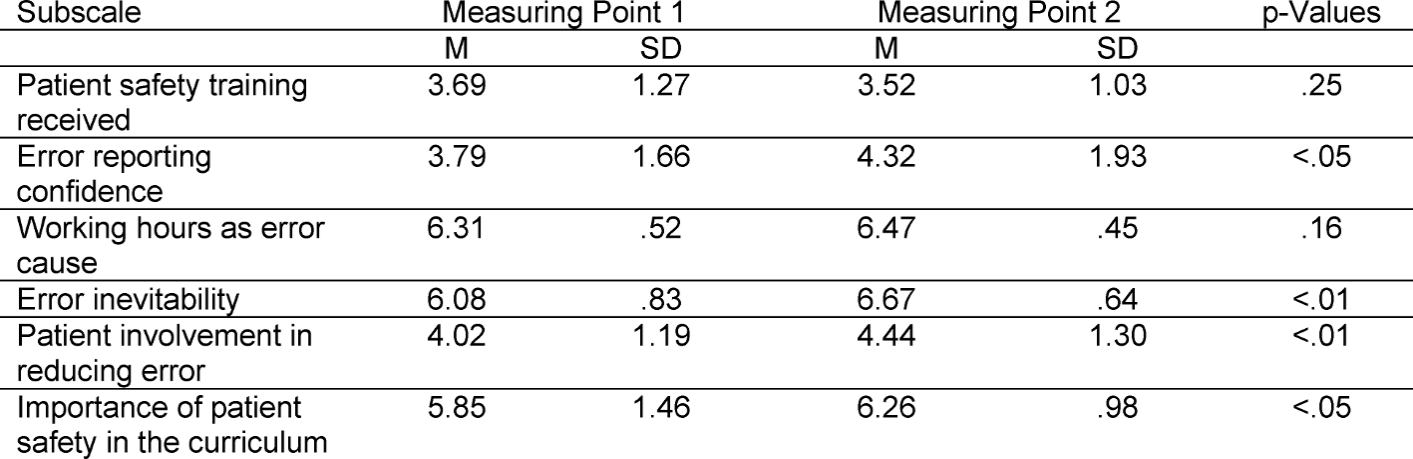
Study 2 descriptive statistics for all subscales for both measuring points. The p-values refer to the post-hoc LSD tests of the MANOVA between both measuring points.
