# Supplementary material for: Validation of a German short version of the Attitudes towards Patient Safety Questionnaire (G-APSQshort) for the measurement of undergraduate medical students' attitudes to and needs for patient safety
Source: GMS J Med Educ. 2017 Feb 15;34(1):Doc8. doi: 10.3205/zma001085 (PMC5327660; doi:10.3205/zma001085)
Supplement: German Short Version of the Attitudes to Patient Safety Questionnaire G-APSQshort [file JME-34-8-s-001.pdf]

German Short Version of the Attitudes to Patient Safety Questionnaire

G-APSQ*short*

Kiesewetter J., Kager M., Fischer M.R., Kiesewetter I.

My training is preparing me to understand the causes of medical errors.	**strongly disagree**	① ② ③ ④ ⑤ ⑥ ⑦	**strongly agree**
I have a good understanding of patient safety issues as a result of my undergraduate medical training.	**strongly disagree**	① ② ③ ④ ⑤ ⑥ ⑦	**strongly agree**
My training is preparing me to prevent medical errors.	**strongly disagree**	① ② ③ ④ ⑤ ⑥ ⑦	**strongly agree**
I would feel comfortable reporting any errors I had made, no matter how serious the outcome had been for the patient.	**strongly disagree**	① ② ③ ④ ⑤ ⑥ ⑦	**strongly agree**
I would feel comfortable reporting any errors other people had made, no matter how serious the outcome had been for the patient.	**strongly disagree**	① ② ③ ④ ⑤ ⑥ ⑦	**strongly agree**
Shorter shifts for doctors will reduce medical errors.	**strongly disagree**	① ② ③ ④ ⑤ ⑥ ⑦	**strongly agree**
By not taking regular breaks during shifts doctors are at an increased risk of making errors.	**strongly disagree**	① ② ③ ④ ⑤ ⑥ ⑦	**strongly agree**
The number of hours doctors work increases the likelihood of making medical errors.	**strongly disagree**	① ② ③ ④ ⑤ ⑥ ⑦	**strongly agree**
Even the most experienced and competent doctors make errors.	**strongly disagree**	① ② ③ ④ ⑤ ⑥ ⑦	**strongly agree**
Human error is inevitable.	**strongly disagree**	① ② ③ ④ ⑤ ⑥ ⑦	**strongly agree**
Patients have an important role in preventing medical errors.	**strongly disagree**	① ② ③ ④ ⑤ ⑥ ⑦	**strongly agree**
Encouraging patients to be more involved in their care can help to reduce the risk of medical errors occurring.	**strongly disagree**	① ② ③ ④ ⑤ ⑥ ⑦	**strongly agree**
Teaching students about patient safety should be an important priority in medical students training.	**strongly disagree**	① ② ③ ④ ⑤ ⑥ ⑦	**strongly agree**
Learning about patient safety issues before I qualify will enable me to become a more effective doctor.	**strongly disagree**	① ② ③ ④ ⑤ ⑥ ⑦	**strongly agree**

Subscale Composition

The instrument is composed of the following subscales. The results are analyzed analogous to the original instrument by calculating the mean and standard deviation of each subscale. When using the instrument the authors kindly asked for citation.

**Patient safety training received**	
	My training is preparing me to understand the causes of medical errors.
	I have a good understanding of patient safety issues as a result of my undergraduate medical training.
	My training is preparing me to prevent medical errors.
**Error reporting confidence**	
	I would feel comfortable reporting any errors I had made, no matter how serious the outcome had been for the patient.
	I would feel comfortable reporting any errors other people had made, no matter how serious the outcome had been for the patient.
**Working hours as error cause**	
	Shorter shifts for doctors will reduce medical errors.
	By not taking regular breaks during shifts doctors are at an increased risk of making errors.
	The number of hours doctors work increases the likelihood of making medical errors.
**Error inevitability**	
	Even the most experienced and competent doctors make errors.
	Human error is inevitable.
**Patient involvement in reducing error**	
	Patients have an important role in preventing medical errors.
	Encouraging patients to be more involved in their care can help to reduce the risk of medical errors occurring.
**Importance of patient safety in the curriculum**	
	Teaching students about patient safety should be an important priority in medical students training.
	Learning about patient safety issues before I qualify will enable me to become a more effective doctor.
