# Supplementary material for: Validation of a German short version of the Attitudes towards Patient Safety Questionnaire (G-APSQshort) for the measurement of undergraduate medical students' attitudes to and needs for patient safety
Source: GMS J Med Educ. 2017 Feb 15;34(1):Doc8. doi: 10.3205/zma001085 (PMC5327660; doi:10.3205/zma001085)
Supplement: Factor loadings of items and their distribution on the 6 factors [file JME-34-8-s-002.pdf]

	Component						
	1	2	3	4	5	6	7
My training is preparing me to understand the causes of medical errors.	,808	-,176			,141	,204	
I have a good understanding of patient safety issues as a result of my undergraduate medical training.	,848	,153		,110			-,107
My training is preparing me to prevent medical errors.	,788	-,152		,108		-,121	
I would feel comfortable reporting any errors I had made, no matter how serious the outcome had been for the patient.		,779	,117	,160	-,191	,167	-,132
I would feel comfortable reporting any errors other people had made, no matter how serious the outcome had been for the patient.		,865					
Shorter shifts for doctors will reduce medical errors.		,523	,207	,120			-,594
By not taking regular breaks during shifts doctors are at an increased risk of making errors.		,105	,912		-,104		
The number of hours doctors work increases the likelihood of making medical errors.			,901		,101		
Even the most experienced and competent doctors make errors.		,159	,833				
Human error is inevitable.			,150	,582	,149	,301	
Patients have an important role in preventing medical errors.	,213	-,136	,367	,612	,152	,189	
Encouraging patients to be more involved in their care can help to reduce the risk of medical errors occurring.			,196		,807	-,175	
Teaching students about patient safety should be an important priority in medical students training.		,296			,752	-,102	,147
Learning about patient safety issues before I qualify will enable me to become a more effective doctor.						,805	,252
My training is preparing me to understand the causes of medical errors.				-,170	-,128	,660	,263
