# Supplementary material for: Validation of a German short version of the Attitudes towards Patient Safety Questionnaire (G-APSQshort) for the measurement of undergraduate medical students' attitudes to and needs for patient safety
Source: GMS J Med Educ. 2017 Feb 15;34(1):Doc8. doi: 10.3205/zma001085 (PMC5327660; doi:10.3205/zma001085)
Supplement: Eigenvalues of the items before rotation [file JME-34-8-s-003.pdf]

	Eigenvalues before rotation
1	4,06
2	2,91
3	2,19
4	2,03
5	1,69
6	1,41
7	1,33
8	1,24
9	1,14
10	0,96
11	0,87
12	0,74
13	0,69
14	0,62
